# The Zn_12_O_12_ cluster-assembled nanowires as a highly sensitive and selective gas sensor for NO and NO_2_

**DOI:** 10.1038/s41598-017-17673-8

**Published:** 2017-12-13

**Authors:** Yongliang Yong, Xiangying Su, Qingxiao Zhou, Yanmin Kuang, Xiaohong Li

**Affiliations:** 1 College of Physics and Engineering, Henan University of Science and Technology, Luoyang, 471023 People’s Republic of China; 2 Henan Key Laboratory of Photoelectric Energy Storage Materials and Applications, Henan University of Science and Technology, Luoyang, 471023 People’s Republic of China; 3 Institute of Photobiophysics, School of Physics and Electronics, Henan University, Kaifeng, 475004 People’s Republic of China

## Abstract

Motivated by the recent realization of cluster-assembled nanomaterials as gas sensors, first-principles calculations are carried out to explore the stability and electronic properties of Zn_12_O_12_ cluster-assembled nanowires and the adsorption behaviors of environmental gases on the Zn_12_O_12_-based nanowires, including CO, NO, NO_2_, SO_2_, NH_3_, CH_4_, CO_2_, O_2_ and H_2_. Our results indicate that the ultrathin Zn_12_O_12_ cluster-assembled nanowires are particularly thermodynamic stable at room temperature. The CO, NO, NO_2_, SO_2_, and NH_3_ molecules are all chemisorbed on the Zn_12_O_12_-based nanowires with reasonable adsorption energies, but CH_4_, CO_2_, O_2_ and H_2_ molecules are only physically adsorbed on the nanowire. The electronic properties of the Zn_12_O_12_-based nanowire present dramatic changes after the adsorption of the NO and NO_2_ molecules, especially their electric conductivity and magnetic properties, however, the other molecules adsorption hardly change the electric conductivity of the nanowire. Meanwhile, the recovery time of the nanowire sensor at T = 300 K is estimated at 1.5 μs and 16.7 μs for NO and NO_2_ molecules, respectively. Furthermore, the sensitivities of NO and NO_2_ are much larger than that of the other molecules. Our results thus conclude that the Zn_12_O_12_-based nanowire is a potential candidate for gas sensors with highly sensitivity for NO and NO_2_.

## Introduction

Since sensing gas molecules is critical to environmental monitoring, control chemical processes, space missions, and agricultural and medical applications, much research has been focused on the development of suitable gas-sensitive materials^1–5^. In recent years, one-dimensional (1D) nanostructured materials have been regarded as one of the most exciting materials for developing new sensing materials and devices due to their excellent properties such as high surface-to-volume ratio, good chemical and thermal stabilities under different operating conditions^5–19^. One of the most common 1D nanomaterials that has been used as gas sensors is zinc oxide (ZnO) nanowires^12–23^.

ZnO nanowires have attracted much interest in the past decade because of their various remarkable physical properties and potential applications not only in gas sensors but also in a number of emerging areas such as low-voltage and short-wavelength optoelectronics, photonics, and solar cells^24–29^. It is well known that the major advantage of using nanowires as gas sensors is the surface-to-volume ratio. That is to say, when the diameter of a nanowire is smaller, its surface-to-volume ratio would be higher, which lead to the electrical properties of the nanowires significantly more sensitive to the specific gas molecules^9^. Moreover, quantum confinement effects in ZnO nanowires are only expected for extremely small diameters as the bulk ZnO exciton Bohr radius of about 2.34 nm^30^, and the potential applications of ZnO nanowires as quantum electron devices require their radial confinement^31^. Therefore, the nanowires diameter is a key parameter in order to realize devices exploiting the quantum confinement effect in ZnO nanowires.

Although ZnO nanowires can be synthesized by many approaches, such as physical/chemical vapor deposition^32–34^ and wet chemical processes^31,35,36^, high-quality ZnO nanowires with much smaller diameters still remain difficult to be synthesized. As we know, only few studies reported on the synthesis of ultrathin ZnO nanowires^32,37–39^. For example, Yin *et al*.^37^ have reported the synthesis of ultrathin, single-crystalline ZnO nanowires with an average diameter of 6 nm, which used micellar gold nanoparticles as catalyst templates. Stichtenoth *et al*.^32^ also reported that the diameter of ultrathin ZnO nanowires can be down to about 4 nm.

In recent years, a “from the bottom up” approach of forming new materials (so-called cluster-assembled materials) of nanoscale dimensions using clusters as building blocks has been developed^40–47^. This approach has opened the pathway to accomplishing the synthesis of new nanoscale materials with tailed properties, indicating that the ZnO nanowires with unusual properties can be assembled by stable ZnO clusters. ZnO clusters have been extensively investigated theoretically and experimentally, and previous reports have demonstrated that Zn_12_O_12_ cluster, as the smallest magic fullerene-like cluster, can be taken as a good candidate for the ideal building blocks for forming cluster-assembled materials^48–55^. Because of the diameter of fullerene-like Zn_12_O_12_ cluster, which is defined as the distance between the most remote two atoms in cluster, is quite small (about 6.4 Å). As a consequence, the diameter of the ZnO nanowires which are assembled by Zn_12_O_12_ cluster would be small, and their properties would be different from that of the traditional ZnO nanowires.

In this work, using first-principles calculations, we firstly demonstrated the feasibility of forming cluster-assembled nanowires based on Zn_12_O_12_ cluster by investigating their structural stabilities and electronic properties. Then, we investigated the adsorption behaviors and electronic properties of gas molecules (including CO, NO, NO_2_, SO_2_, NH_3_, CH_4_, CO_2_, O_2_, and H_2_) on the Zn_12_O_12_-based nanowires, to find out the possibility of using the Zn_12_O_12_-based nanowires as gas sensors for some certain gases detection.

## Computational Methods

The spin-polarized density functional theory (DFT) implemented in the DMol^3^ program^56,57^ was performed for structural relaxation and electronic calculations. The generalized gradient approximation formulated by Perdew, Burke, and Ernzerhof (PBE)^58^ with van der Waals (vdW) correction proposed by Tkatchenko and Scheffler (TS method)^59^ was chosen to describe the exchange-correlation energy functional. Density-functional semi-core pseudopotentials (DSPPs)^60^ fitted to all-electron relativistic DFT results, and double numerical basis sets supplemented with *d* polarization functions (i.e. the DND set) were selected. The charge transfer was analyzed based on Hirshfeld analysis^61^, which is based directly on the electron density as a function of space. A tetragonal supercell of 20 × 20 × *L* Å^3^ was set for all calculations, where *L* is the length of translational periodicity. The Brillouin zone was sampled by 1 × 1 × 10 special *k*-points for structural relaxation, while 1 × 1 × 15 *k*-points for electronic structure calculations using the Monkhorst-Pack scheme^62^. In our previous studies, we have confirmed the reliability of the GGA-PBE and DND combination for predicting structural and electronic properties of (doped) ZnO clusters and cluster-assembled materials^50,63,64^.

The binding energy per ZnO (*E*_b_) is defined as

1 $${E}_{{\rm{b}}}=(n{E}_{{\rm{Zn}}}+n{E}_{{\rm{O}}}-{E}_{({\rm{ZnO}})n})/n,$$

where *E*_Zn_, *E*_O_, and *E*_(ZnO)*n*_ are the total energies of an isolated Zn atom, an isolated O atom, the corresponding (ZnO)_*n*_ system, respectively, and *n* is the number of Zn or O atoms involved. To investigate the stability of the adsorption of molecules on Zn_12_O_12_-based nanowires, the adsorption energy (*E*_ads_) is defined as

2 $${E}_{{\rm{ads}}}={E}_{({\rm{nanowire}}+{\rm{molecule}})}-{E}_{({\rm{nanowire}})}-{E}_{({\rm{molecule}})},$$

where *E*_(nanowire+molecule)_, *E*_(nanowire)_ and *E*_(molecule)_ is the total energy of the system of molecule adsorbed on the nanowire, the corresponding pure nanowire and molecule, respectively.

## Results and Discussion

### The cluster-assembled nanowires based on Zn_12_O_12_

**Figure 1:**
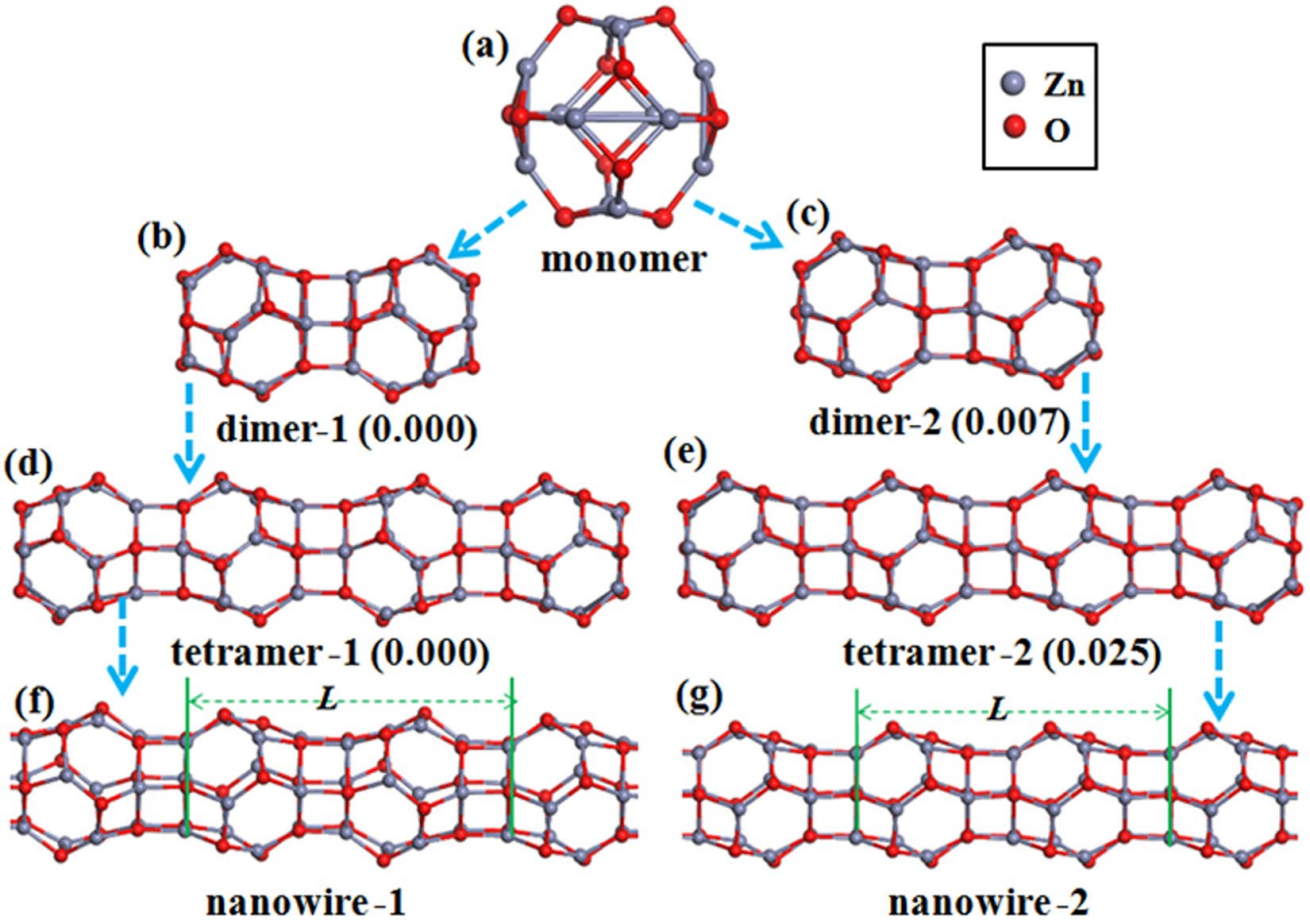
The optimized configurations of the Zn_12_O_12_-based nanostructures: (**a**) the fullerene-like Zn_12_O_12_ cluster; (**b** and **c**), the two most stable structures of Zn_12_O_12_ dimers; (**d** and **e**), the two most stable structures of Zn_12_O_12_ tetramers; (f and g), the Zn_12_O_12_-based nanowires. Values in parentheses (in eV) are relative energies with respect to the most stable isomer for each composition. “*L*” as shown in figures is the length of translational periodicity for the optimized nanowires, 13.671 and 11.689 Å for nanowire-1 and nanowire-2, respectively.

Recently, Zn_12_O_12_ cluster has been probed by time-resolved photoelectron spectroscopy^52,53^, which further demonstrates that Zn_12_O_12_ cluster is a promising building block for cluster-assembled materials with tailed properties. In previous work, we have proposed one growth path of Zn_12_O_12_ cluster-cluster coalescence, and predicted that assembly can form by attaching a Zn_12_O_12_ cage on a hexagonal site^50^. Further, Liu *et al*.^51^ have characterized a family of Zn_12_O_12_ cluster-assembled solid phases with novel structures and properties, and the most phases are formed by the hexagonal face coalescence. It is well known that Zn_12_O_12_ cluster is a highly stable cluster with a cage structure with six isolated four-membered rings (4MRs) and eight six-membered rings (6MRs), which is shown in Fig. 1(a). Two most stable Zn_12_O_12_ dimers are presented in Fig. 1(b and c). It can be seen that both two dimers are formed by the hexagonal face coalescence. However, what is different is that in the most stable one a 6MR of one monomer is connected with a 6MR of the other monomer, while in the second stable one a 6MR of one monomer is connected with a 4MR of the other monomer. Although the structures of the two dimers are different, the energy difference is very small (only 0.007 eV). These results are in good agreement with previous work^51^. On the basis of the two coalescence ways shown in dimers, we further investigated the Zn_12_O_12_ tetramers. It is found that the wire-like structure of tetramers can be viewed as the coalescence of the two corresponding dimers, which is shown in Fig. 1(d and e), further indicating that the wire-like structures can continue. To investigate the relative stability of Zn_12_O_12_ dimers and tetramers, we calculated the binding energy per ZnO (*E*_b_) and dimerization energy per unit (A Zn_12_O_12_ as an unit) (*E*_d_), which is defined as

3 $${E}_{{\rm{d}}}=(n{E}_{{\rm{m}}}-{E}_{{\rm{t}}})/n,$$

where *E*_m_ and *E*_t_ are the total energies of Zn_12_O_12_ monomer and the corresponding dimer (or tetramer), respectively, and *n* is the number of monomers. These results are listed in Table 1. It is found that the values of *E*_b_ and *E*_d_ of Zn_12_O_12_ monomer, dimer, tetramer, and nanowire follow the rules: nanowire > tetramer > dimer > monomer, indicating that the (Zn_12_O_12_)_n_ assemblies are more stable than (Zn_12_O_12_)_n-m_ assemblies (n > m), which is similar to the cases of other M_12_N_12_ clusters-assembled materials^65,66^. Furthermore, the HOMO-LUMO gaps of dimers and tetramers are similar to the Zn_12_O_12_ monomer, which further indicates that the basic properties of the isolated Zn_12_O_12_ monomer can retain during the cluster assembling.

**Table 1:** The calculated binding energy per ZnO (*E*_b_), dimerization energy per unit (*E*_d_), and HOMO-LUMO gap (or band energy gap) (*E*g) for the Zn_12_O_12_-based nanostructures.

System	*E*_b_ (eV)	*E*_d_ (eV)	*E*_g_ (eV)
monomer	6.356	—	2.529
dimer-1	6.541	2.225	2.225
dimer-2	6.540	2.221	2.283
tetramer-1	6.635	3.345	2.151
tetramer-2	6.634	3.338	2.205
nanowire-1	6.723	4.400	2.106
nanowire-2	6.722	4.390	2.159

Based on the coalescence ways and characters of the dimers and tetramers, we can conclude that the Zn_12_O_12_-based nanowires can be formed by a translational symmetry arrangement. The segments of the two stable configurations of Zn_12_O_12_-based nanowires are shown in Fig. 1(f and g), named as nanowire-1 and nanowire-2, respectively. The calculated binding energy per ZnO (*E*_b_) and the band energy gap are summarized in Table 1. It is clear that the structural geometry of the isolated Zn_12_O_12_ monomer can be maintained in the assembled nanowires. This feature is similar to the cases of other cluster-assembled materials based on Zn_12_O_12_^50,51^. From Table 1, it can be seen that the *E*_b_ of the two nanowires are larger than that of monomer, dimers and tetramers, suggesting that the stability of Zn_12_O_12_-based nanostructures is strengthened because of the assembly continuing. The stability of the above discussed nanostructures (monomer, dimer, tetramer, and nanowire) is also demonstrated by their vibrational frequencies calculations. We found that these structures have no imaginary frequencies, indicating that they are real stable. The most stable nanowire is the nanowire-1, in which one 6MR of one monomer faces to a 6MR of adjacent monomer, which is the same as the coalescence way in the most stable dimer and tetramer.

**Figure 2:**
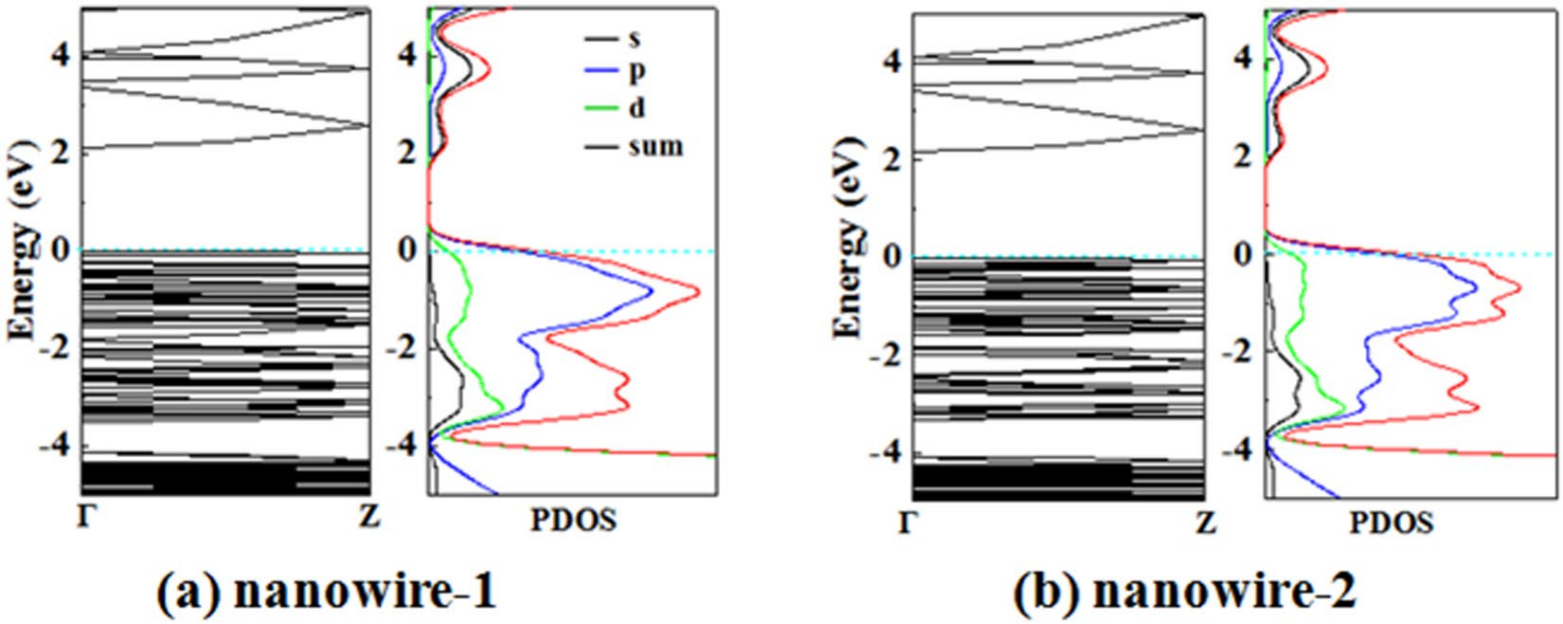
Electronic band structures and total and partial DOS for the two Zn_12_O_12_-based nanowires. The light-blue dashed line is the Fermi-level energy.

The energy band structures for the two Zn_12_O_12_-assembled nanowires have been calculated and were plotted in Fig. 2. A direct gap of 2.102 eV is found for the most stable nanowire named as nanowire-1, whereas a direct gap of 2.159 eV is found for the second stable nanowire named as nanowire-2, indicating that both nanowires have semiconducting electrical properties. The band-gap widths of the both nanowires are much larger than that of the ZnO nanowires that are constructed from a (7 × 7 × 2) ZnO wurtzite supercell containing 96 atoms (Zn_48_O_48_) with 13.011 Å along the $[10\overline{1}0]$ and $[01\overline{1}0]$ directions^67^, which shows that the electronic properties of the two Zn_12_O_12_-assembled nanowires are different from that of the traditional ZnO nanowires with wurtzite structures. Furthermore, the total and partial density of states (DOS) of the two Zn_12_O_12_-assembled nanowires were calculated to reach a deep understanding of the features in the band edges near the band gaps, which are also shown in Fig. 2. The valence bands near Fermi level are mainly contributed by O 2p states, next by Zn 3d states, leading to a significant hybridization of O 2p and Zn 3d levels, while the lowest unoccupied state is mainly dominated by Zn 4 s sates. The features of the DOSs of the Zn_12_O_12_-assembled nanowires are similar to the case of ZnO nanotubes^68^.

**Figure 3:**
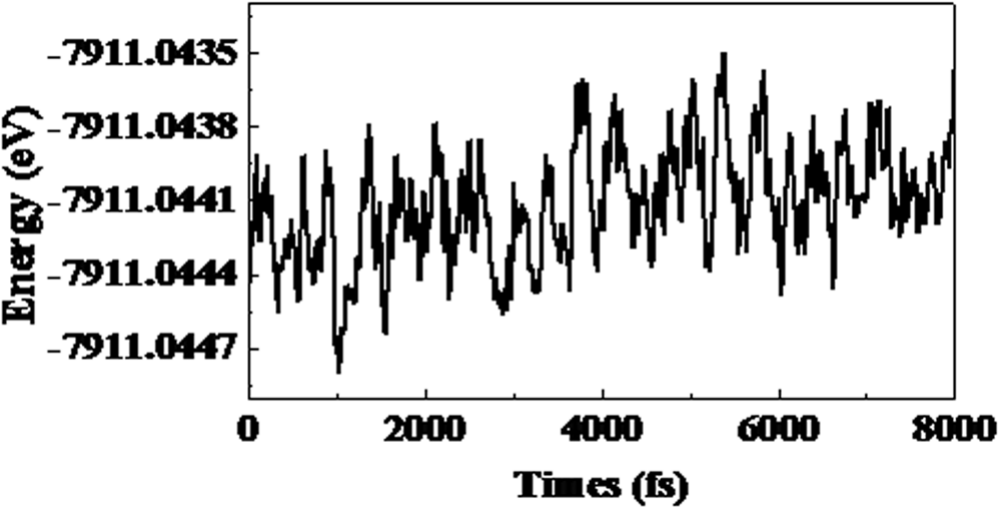
Variation in the energy (eV) of the most stable configuration of the Zn_12_O_12_-based nanowire-1 as a function of time at *T* = 300 K.

Although we have concluded that the two Zn_12_O_12_-assembled nanowires are real stable because of no imaginary frequencies, to develop their potential use as gas sensors, it also should figure out their thermodynamic stability. As a consequence, the thermodynamic stability of the most stable Zn_12_O_12_-based nanowire (i.e. nanowire-1) as an example was investigated using the first-principles Born-Oppenheimer molecular dynamics (BOMD) simulation within a NVT ensemble. The method of GGA-PBE and DND combination in the work was used for BOMD. The temperature was controlled by a Nosé-Hoover chain of thermostats. A simulation time of 8 ps with a time step of 1 fs was set, and the structural change was calculated at a constant temperature of 300 K. The variation in the energy as a function of time is shown in Fig. 3. It can be seen from Fig. 3 that no structural instability was observed at the temperature of 300 K. In addition, the total energies of the considered nanowire-1 during simulations also keep stable oscillation within a quite small range (no more than 0.001 eV), strongly supporting the fact that the structure of nanowire-1 is thermodynamic stable at room temperature. Based on the analysis of binding energy, dimerization energy, vibrational frequencies and thermodynamic stability, it is demonstrated that the assembly of Zn_12_O_12_ cluster can form thermodynamic stable one-dimensional nanowires, which can be used as gas sensing materials. However, it is noting that the assembly of Zn_12_O_12_ cluster is easier to form three-dimensional phases if there is no any extra restriction, which has been predicted by previous work^51,52^. Even so, our results provided a way to form the ultrafine ZnO nanowires through the assembly of Zn_12_O_12_ cluster. For the experimental synthesis of ZnO nanowires by coalescence of Zn_12_O_12_ cluster, the cluster evolution into nanowires can be through templating or aggregative mechanisms^69^, which should be similar to the case of single CdTe nanoparticles (or clusters) evolution into nanowires^70,71^. We then investigated the feasibility of the thermodynamic stable Zn_12_O_12_-based nanowire-1 as a gas sensing material.

### Adsorption of CO, NO, NO_2_, SO_2_, NH_3_, CH_4_, CO_2_, O_2_ and H_2_

**Figure 4:**
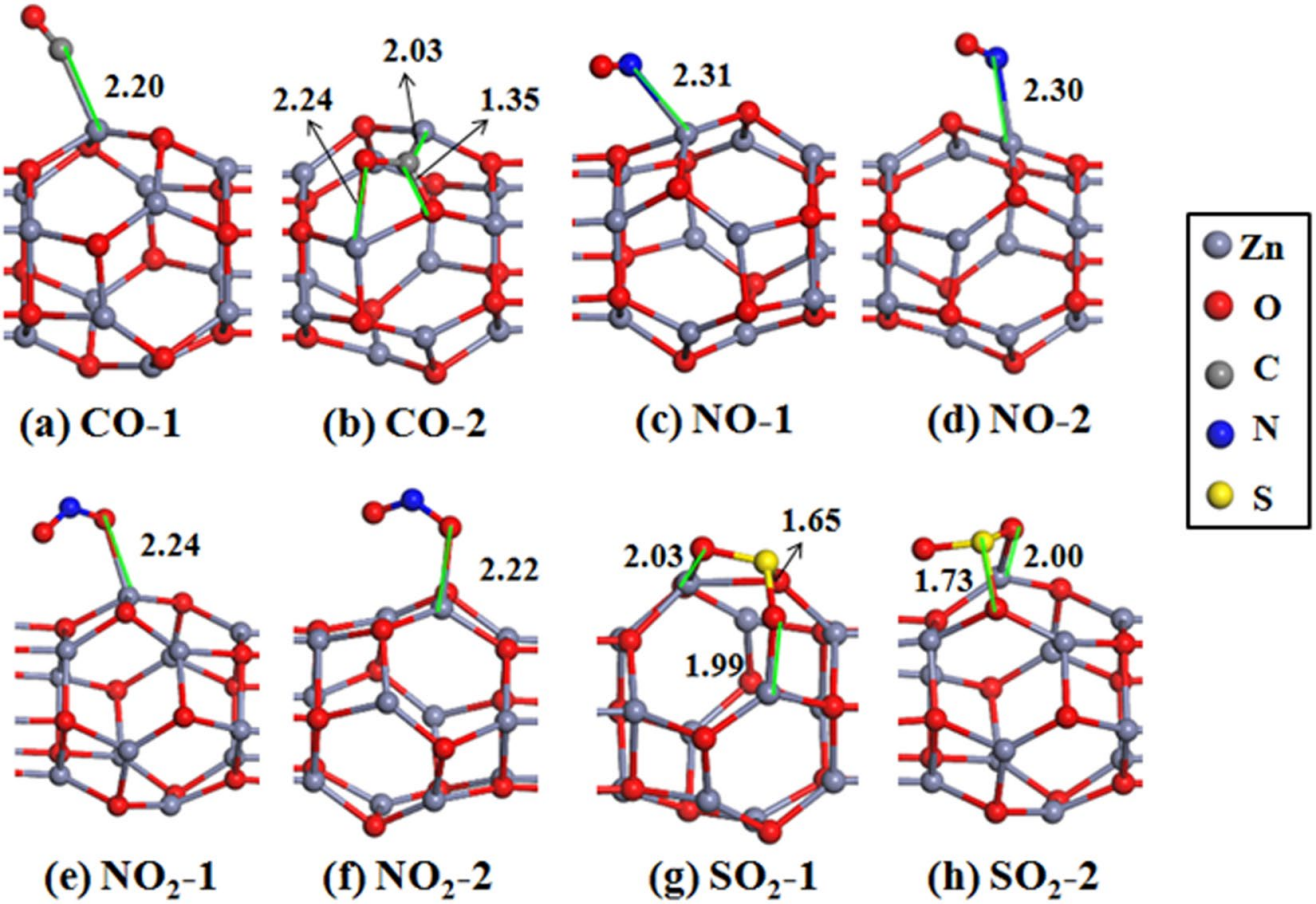
Optimized structures of the Zn_12_O_12_-based nanowire with gas molecule adsorption: (**a**) and (**b**) CO; (**c**) and (**d**) NO; (**e**) and (**f**) NO_2_; (**g**) and (**h**) SO_2_. The structure around the adsorbed molecule is shown in figures. Isomeric structures of each molecule on the nanowire are labeled as molecule-1, molecule-2 etc in order of decreasing stability. The bond lengths (in Å) between the molecule and the nanowire are also given.

**Figure 5:**
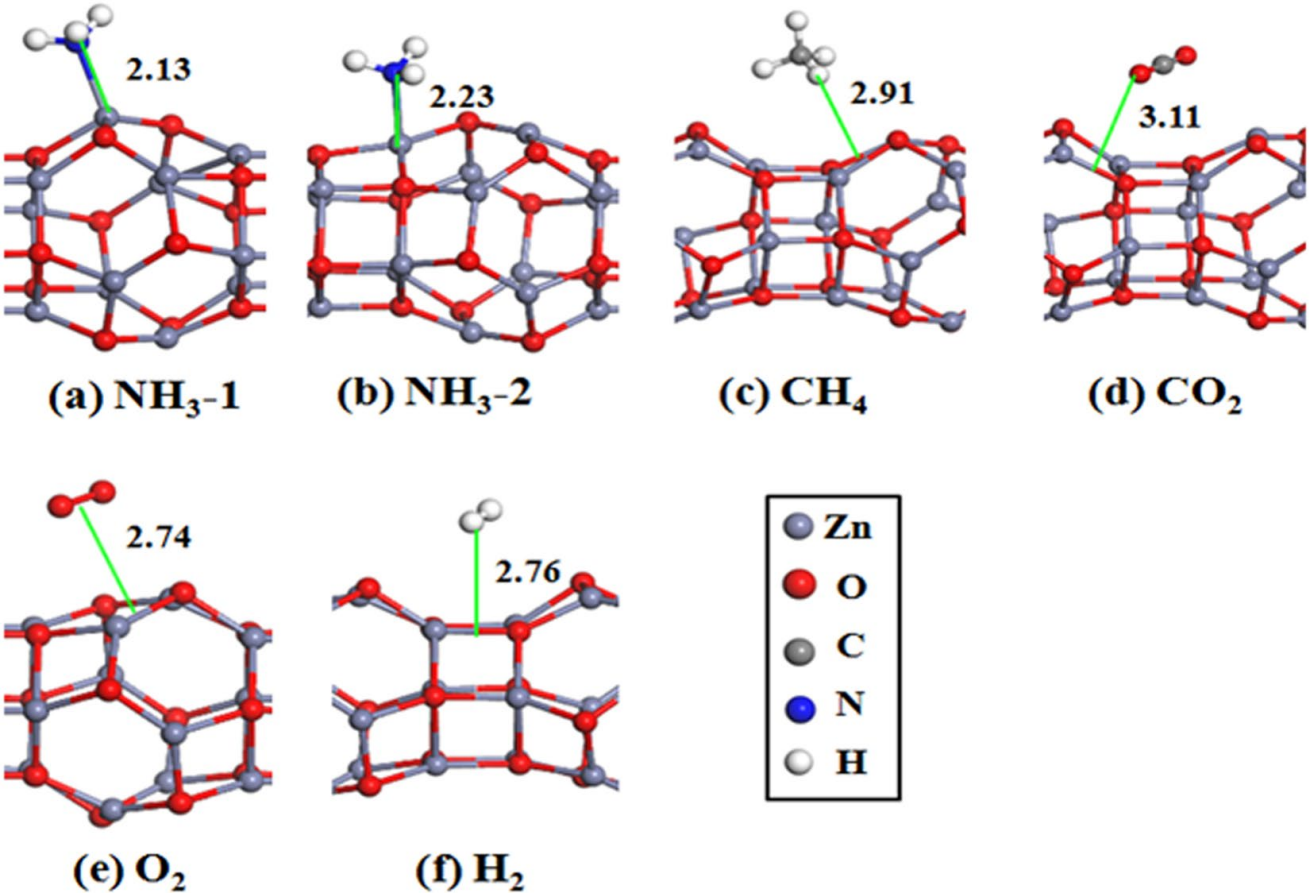
Optimized structures of the Zn_12_O_12_-based nanowire with gas molecule adsorption: (**a**) and (**b**) NH_3_; (**c**) CH_4_; (**d**) CO_2_; (**e**) O_2_ and (**f**) H_2_. The structure around the adsorbed molecule is shown in figures. The bond lengths (in Å) between the molecule and the nanowire are also given.

We started by investigating the adsorption geometries of nine gas molecules on the Zn_12_O_12_-based nanowire. To obtain the most stable configurations of each molecule adsorbed on the Zn_12_O_12_-based nanowire, we have considered the initial structures for the molecule-nanowire systems as many as possible. Figures 4 and 5 show the most stable and some low-lying structures of each molecule on the Zn_12_O_12_-based nanowire, and the corresponding results are listed in Table 2. It is found that different gas molecules prefer different adsorption geometries.

**Table 2:** Calculated adsorption energy (*E*_ads_), charge transfer from the Zn_12_O_12_-based nanowire to molecule (*E*_T_), and the band gap (*E*_g_) for the adsorption of the considered molecules on the Zn_12_O_12_-based nanowire.

system	*E*_ads_ (eV)	*E*_T_ (e)	*E*_g_ (eV)
CO-1	−0.404	0.148	2.100
CO-2	−0.340	−0.212	2.117
NO-1	−0.367	0.075	0.525
NO-2	−0.355	0.051	0.630
NO_2_-1	−0.430	0.020	0.259
NO_2_-2	−0.400	0.031	0.284
SO_2_-1	−1.281	−0.249	2.140
SO_2_-2	−1.072	−0.266	2.160
NH_3_-1	−1.187	0.261	2.096
NH_3_-2	−0.806	0.192	2.080
CH_4_	−0.270	−0.050	2.105
CO_2_	−0.297	0.013	2.098
O_2_	0.221	0.008	0.795
H_2_	0.154	−0.025	2.112

For the CO molecule adsorbed on the nanowire, it is found that the configuration of CO molecule located at the top of one Zn atom is the most stable as shown in Fig. 4(a), and the C atom is bonded with one Zn atom forming a Zn-C bond, whose bond length is 2.20 Å (i.e. adsorption distance). At this configuration the adsorption energy is −0.404 eV. The Zn-C bond length is larger than the average value listed in the Cambridge Structural Database (2.01 Å), but in agreement with that of zinc carbatrane compounds^72^. More importantly, the CO molecule can locate at the top of each Zn atom with little energy difference, compared with the most stable one. The CO molecule is also found that it can absorb on the nanowire with the configuration of the C atom located at the bridge of Zn-O bond, which is shown in Fig. 4(b). Its adsorption energy is −0.340 eV, a little higher (0.064 eV) than that of the most stable one. Similar to the most stable configuration of CO on the nanowire, the NO molecule prefers to locate at the top of an arbitrary Zn atom with the *E*_ads_ of −0.367 ~ −0.355 eV, which is shown in Fig. 4(c–d). Although we have considered a lot of initial structures, no other kind of configurations is found for the NO adsorption.

For the most stable NO_2_ adsorption, one O atom of NO_2_ is bonded with a Zn atom with the bond length of 2.24 Å. Different geometries of NO_2_ adsorption only reflect in the relative location between the molecule and nanowire, see Fig. 4(e–f). This is very similar to the case of NO_2_ adsorption on ZnO nanotubes^11^, but different from the adsorption behaviors of NO_2_ one other M_12_N_12_ cluster-assembled nanowires^65^. Very different from the adsorption of NO_2_, the SO_2_ molecule adopted the orientation of O-S-O parallel to one edge of Zn-O-Zn in the nanowire, and the configuration of SO_2_ located on this edge is the most stable, as shown in Fig. 4(g), meanwhile one Zn-O bond is broken due to the interaction between the SO_2_ and nanowire, which result in a large *E*_ads_ of −1.281 eV, indicating strong interaction between the SO_2_ and the nanowire. The structural deformation of the nanowire may be a limitation for its application as a gas sensor. In the second stable configuration as shown in Fig. 4(h), there are one O atom and S atom bonded with Zn and O atom, respectively. It is 0.209 eV higher in energy than that of the most stable one. For the NH_3_ molecule adsorption, the N atom is directly bonded to one Zn atom with relatively large *E*_ads_ of −1.187 eV. It is noted that for the most stable configuration of NH_3_ adsorption, the bonded Zn atom is not located in the interaction region of Zn_12_O_12_ monomers. When the N atom is bonded to the Zn atom that is located in the interaction region of Zn_12_O_12_ monomers, the interaction between the molecule and nanowire would be weaken, as a matter of fact of the adsorption energy of −0.806 eV.

Based on the analysis of adsorption energy and bonding condition between the molecule and nanowire, it can be concluded that the CO, NO, NO_2_, SO_2_, and NH_3_ molecules are all chemically adsorbed on the Zn_12_O_12_-based nanowire. Moreover, it can be seen from Table 2, because of the chemisorptions of CO, NO, NO_2_, SO_2_, and NH_3_, there are obvious charge transfers between the molecules and the Zn_12_O_12_-based nanowire, especially for CO, SO_2_, and NH_3_ adsorption. Though the adsorption of NO and NO_2_ result in very small charge transfers (less than 0.1 *e*), it should be noted that our results are obtained using the Hirshfeld method. Actually, almost all charge schemes give significantly larger atomic charges than Hirshfeld scheme, as a consequence, Hirshfeld charges are too small^73^.

Besides the above gas molecules, we also study the adsorption of CH_4_, CO_2_, O_2_ and H_2_ molecules on the Zn_12_O_12_-based nanowire. The most stable configurations of CH_4_, CO_2_, O_2_ and H_2_ on the nanowire are shown in Fig. 5(c–f), respectively. It is found that these molecules are all physically adsorbed on the nanowire with quite small adsorption energies and charge transfers, especially for O_2_. It is difficult for O_2_ to adsorb on the Zn_12_O_12_-based nanowire, indicating that the high inertness of the Zn_12_O_12_-based nanowire toward O_2_.

**Figure 6:**
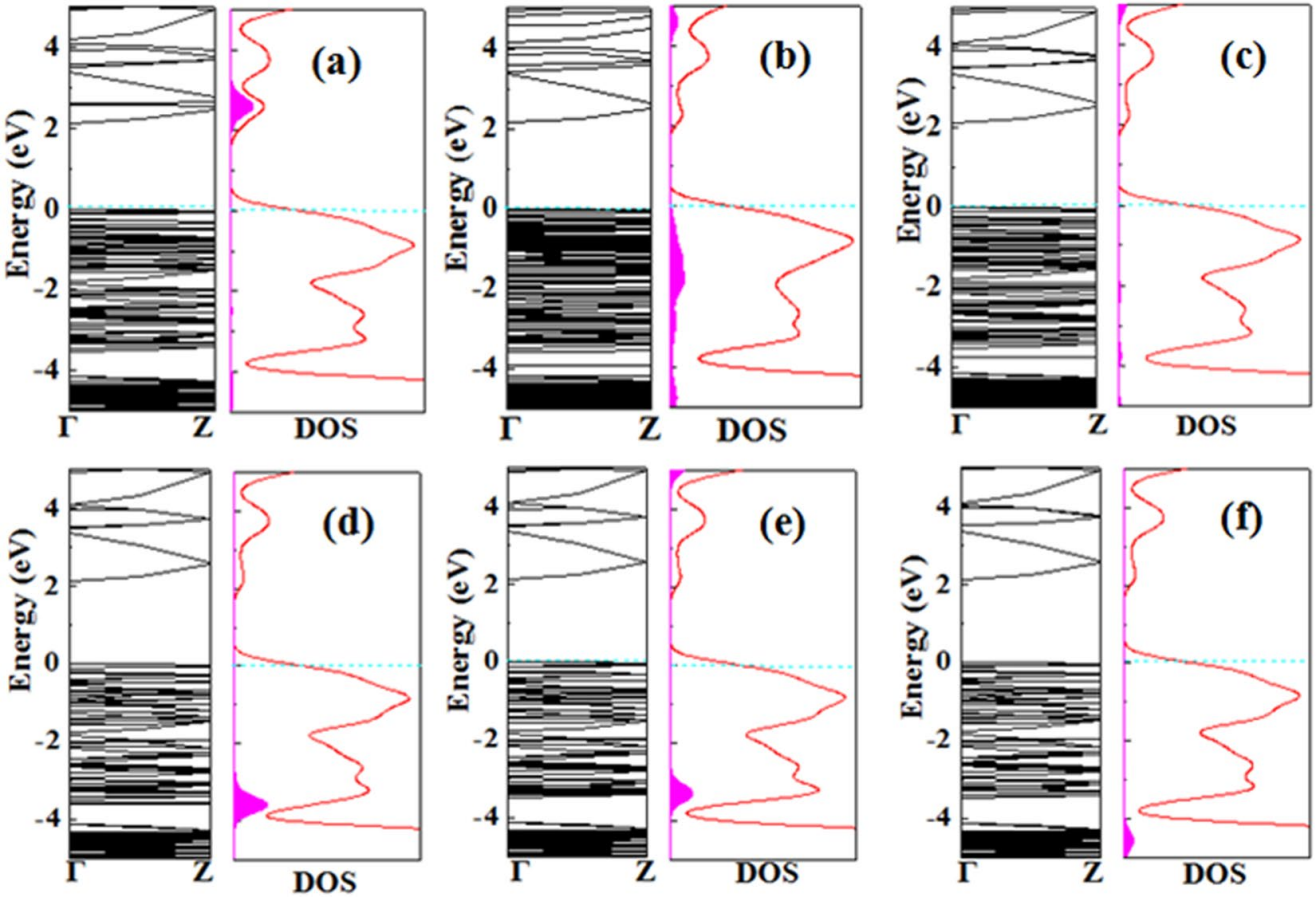
Electronic band structures and density of states (DOS) of the Zn_12_O_12_-based nanowire with gas molecule adsorption: (**a**) CO, (**b**) SO_2_, (**c**) NH_3_, (**d**) CH_4_, (**e**) CO_2_, and (**f**) H_2_. The LDOS of the corresponding gas molecules are also plotted and indicated by purple-red area in DOS curve. The Fermi level is set to zero and indicated by Ocean-blue dashed lines.

**Figure 7:**
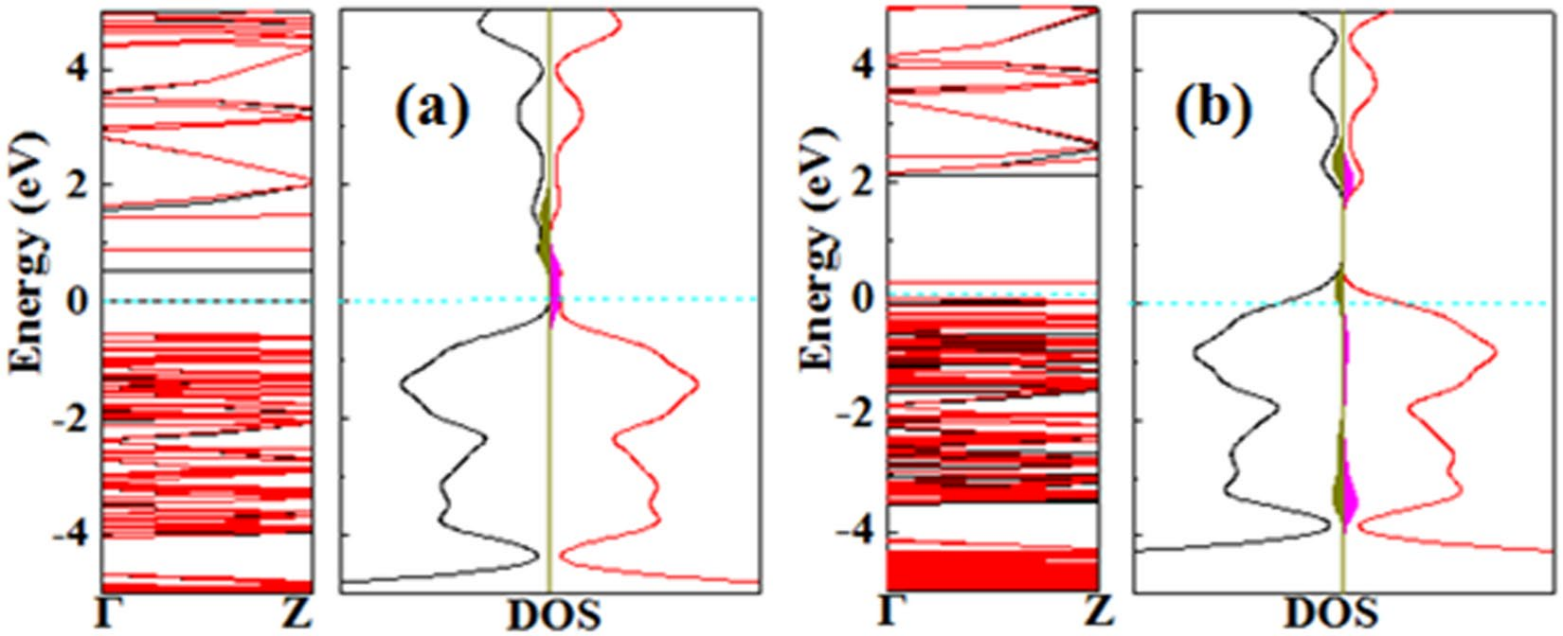
Electronic band structures and density of states (DOS) of the most stable configurations of (**a**) NO and (**b**) NO_2_ molecules adsorbed on the Zn_12_O_12_-based nanowire. Spin-up and spin-down states are shown in black and red lines, respectively. The LDOS of gas molecules is plotted as purple-red (or chrome-green) area in DOS curve. The Fermi level is set to zero and indicated by Ocean-blue dashed lines.

We now turn to investigate the influence of gas adsorption on the electronic properties of the Zn_12_O_12_-based nanowire. Electronic band structures and total density of states (DOS) for the most stable configurations of each molecule adsorbed on the Zn_12_O_12_-based nanowire as well as local DOS (LDOS) of the corresponding molecules are shown in Figs 6 and 7, and the corresponding band-gap widths are summarized in Table 2. Compared with the band structures and DOS of the pure Zn_12_O_12_-based nanowire, it can be seen that the adsorption of a single CO, NH_3_, CH_4_, CO_2_, and H_2_ molecule per supercell does not introduce any impurity states in the band gap, resulting in a little change of the band gap widths. Similar results were also reported on the adsorption of CO, H_2_ and NH_3_ molecules on the $(10\overline{1}0)$ ZnO surface^74^ and nanotubes^11^. Although the charge transfer from the CO molecule to the nanowire does lead the CO molecule to introduce some impurity states within the conduction band, which is located at about 2.5 eV above the Fermi level, it is not expected that the adsorption of NH_3_, CH_4_, CO_2_, and H_2_ molecules hardly enhance the electronic conductance of the Zn_12_O_12_-based nanowire, which would block the applications of the Zn_12_O_12_-based nanowires as gas sensors for CO, NH_3_, CH_4_, CO_2_, and H_2_ detection. For the adsorption of the SO_2_ molecule as shown in Fig. 6(b), LDOS analysis shows that SO_2_ adsorption will produce fully occupied states, which are strongly hybridized with the original “bulk” states in the valence bands, and these states are nonlocalized. These results indicate that the interaction between the SO_2_ molecule and the nanowire is very strong, which is in agreement with the biggest adsorption energy (−1.281 eV). It is very difficult for the fully occupied states resulted from SO_2_ to influence the electronic conductance of the Zn_12_O_12_-based nanowire. From the above analysis, it can be concluded that, although CO, SO_2_, and NH_3_ gases are chemisorbed on the nanowire with reasonable *E*_ads_ and apparent charge transfer, the electronic properties, especially the electronic conductance of the nanowire indeed are not influenced by the adsorption of CO, SO_2_, and NH_3_. This may indicate that the charge transfer does not affect the electronic conductivity of the nanowire, which is very different from that of ZnO surfaces^74^.

However, for the adsorption of NO and NO_2_ molecules, it is found that these both molecules adsorption does introduce impurity states in the band gap, and especially some certain unoccupied local states in the conduction as shown in Fig. 7, resulting in the decrease of the original band gap. More importantly, the Fermi level is shifted to original conduction bands for NO adsorption. From this perspective, we do expect that these impurity states and unoccupied local states that produced by NO molecule adsorption can change the conductance of the Zn_12_O_12_-based nanowire. Although the mid-gap states produced by NO_2_ may not change the conductance of the Zn_12_O_12_-based nanowire, as mentioned above, NO_2_ (the same as NO) behaves as a charge acceptor because of the charge transfer (0.02 e) from the nanowire to the molecule. The charge transfer behavior may influence the electronic conductance of the Zn_12_O_12_-based nanowire, which would be consistent with the experimental findings in that the electronic conductance of ZnO nanowires changes when exposed to NO_2_^13^.

It is known that DFT-GGA not only greatly underestimates the band gaps of bulk ZnO, but also of low-dimensional ZnO structures^11,17^, and the hybrid functional or DFT + *U* can give a quantitatively more accurate band gaps of the ZnO structures^11,17^. However, previous work have found that the overall features of the band structures calculated by using different hybrid functional or DFT + *U* are nearly the same as that based on the DFT-GGA method^11,17,51,67,68^. Thus, we used the DFT-HSE06 hybrid functional^75^ to recalculate the band structures of the most stable configuration of the Zn_12_O_12_-based nanowire without and with NO adsorption, respectively (see Supporting Information Figure S1). The calculated band gaps of the Zn_12_O_12_-based nanowire without and with NO adsorption are 3.438 and 1.110 eV, respectively, much larger than that obtained from GGA-PBE. Compared with the band structures that calculated by GGA-PBE, one can find that the two functional predict similar dispersion features for both valence and conduction bands. These results are in agreement with previous work^11,17,51,67,68^. Since both DFT-HSE06 and GGA-PBE calculations show that the band gap of the Zn_12_O_12_-based nanowire is larger than that of the bulk ZnO (the calculated band gap value of 0.94 eV), we may expect that the qualitatively correct trend in the band structure can still be drawn from the GGA-PBE calculation. More importantly, as discussed below, the results are valuable for comparative studies on the change of band gaps because of the molecule adsorption.

### The possibility of the Zn_12_O_12_-based nanowire as gas sensors

In general, a good commercial sensor should face the following challenges: sensor sensitivity, selectivity, stability, and speed (response and recovery rate), namely the “4 s”. Next, we would discuss the possibility of the Zn_12_O_12_-based nanowire as a gas sensor for some certain gas detection, that is, whether the gas sensors based on the Zn_12_O_12_-based nanowire can exhibit better performances.

As mentioned above, the Zn_12_O_12_-based nanowire is particularly thermodynamic stable at room temperature. After the NO and NO_2_ molecules adsorption, it is found that there is no structural deformation in Zn_12_O_12_-based nanowire, further indicating the stability of Zn_12_O_12_-based nanowire for the adsorption of NO and NO_2_.

If the Zn_12_O_12_-based nanowire is indeed effective as a gas sensor for certain gas detection, the molecular gases would be chemically adsorbed on the Zn_12_O_12_-based nanowire with apparent *E*_ads_. As above discussed, CO, NO, NO_2_, SO_2_, and NH_3_ molecules are all chemisorbed on the nanowire with reasonable *E*_ads_, but CH_4_, CO_2_, O_2_ and H_2_ molecules are only physically adsorbed on the nanowires with small *E*_ads_ and little charge transfer, indicating that the nanowire is incapable for sensing CH_4_, CO_2_, O_2_ and H_2_ gases. Furthermore, the conductivity under a certain temperature can be estimated by the following equation:

4 $$\sigma \propto \exp (\frac{-{E}_{g}}{kT}),$$

where *σ* is the electric conductivity of the configurations, *E*_g_ is the band gap value of the configurations, *k* is the Boltzmann’s constant, and *T* is the thermodynamic temperature. As the equation is shown that the conductivity is controlled by exp (−*E*_g_/*kT*) under a certain temperature, a change of electronic conductance would be observed by experiment. Therefore the changes of electronic conductance of the nanowire before and after the adsorption can be used to differentiate the molecules because of the change of *E*_g_. It is found that the adsorption of CO, SO_2_, and NH_3_ molecules makes the change of band gap of Zn_12_O_12_-based nanowire be very small, indicating the adsorption of these molecules hardly has any impact on the electric conductivity of the nanowire. This is consisting with the analysis of band structures and DOS. However, the adsorption of NO and NO_2_ changes the band gap widths of the nanowire as obvious as from 2.106 eV for pure nanowire to 0.525 and 0.259 eV, respectively. Therefore, the NO and NO_2_ molecules can be detected by calculating the conductivity change in the Zn_12_O_12_-based nanowire before and after the adsorption process.

**Figure 8:**
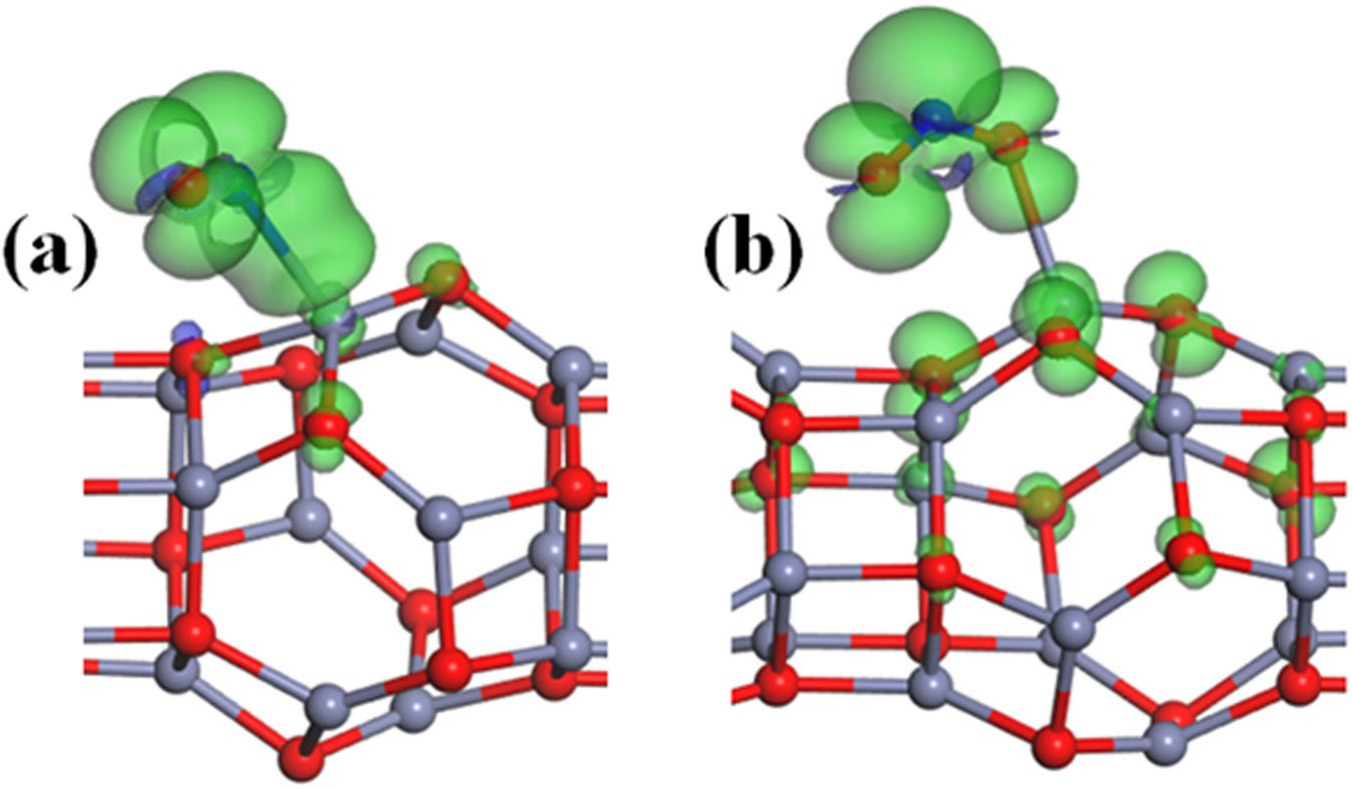
Spin density of the most stable configurations of (**a**) NO and (**b**) NO_2_ adsorption on the Zn_12_O_12_-based nanowire with isovalues of ±0.008 e/Å^3^.

If the Zn_12_O_12_-based nanowire cannot detect the NO and NO_2_ molecules because of the change of conductance, it also can be concluded by using a completely new transduction principle, which is the exploitation of magnetic instead of electrical properties modifications due to surface–gas interaction in the active material^6,76^. Our results show that the Zn_12_O_12_-based nanowire is non-magnetic. The spin density of the most stable configurations of NO_2_ (or NO) adsorbed on the Zn_12_O_12_-based nanowire is shown in Fig. 8. The adsorption of NO (or NO_2_) molecule introduces spin polarization in the Zn_12_O_12_-based nanowire with a magnetic moment of approximately 1 *μ*_*B*_, indicating that magnetic properties of the Zn_12_O_12_-based nanowire is changed obviously due to the adsorption of NO (or NO_2_) molecule. Furthermore, the magnetic moment is mainly located at the NO_2_ (or NO) molecule, and chiefly originates from the 2p states of N and O atoms. However, the net spin polarization of the Zn_12_O_12_-based nanowire is not modified due to the adsorption of the other considered molecules. In this regard of the change of magnetic properties, the Zn_12_O_12_-based nanowire can be viewed as a highly sensitive gas detection technique based on the measurement of the local magnetic moment in the Zn_12_O_12_-based nanowire using various experimental methods such as AFM or SQUID magnetometry^77–79^.

Furthermore, the strong interaction between the molecules and the Zn_12_O_12_-based nanowire also block the applications of the nanowire as a gas sensor. This is because that strong adsorption would make the molecules desorbed from the nanowire very difficultly, and the devices would suffer a longer recovery time. The transition state theory gives the relation between the recovery time (*τ*) and the adsorption energy (*E*_ads_) as follows:

5 $$\tau ={\nu }_{0}^{-1}{e}^{-{E}_{ads}/kT},$$

where *ν*_0_ is the attempt frequency, *k* is the Boltzmann’s constant, and *T* is the temperature. Supposing all the considered molecules have the same order of magnitude for the attempt frequency as NO_2_ (*ν*_0_ = 10^12^ s^−1^)^80^. We can estimate that the strong adsorption energies of SO_2_ and NH_3_ on the nanowire (more than −1 eV) would match a recovery time of much more than 12 hours at T = 300 K, which further shows that the Zn_12_O_12_-based nanowire is not suitable as a reusable sensor for SO_2_ and NH_3_ gases. Because of the adsorption energy of −0.367 and −0.430 eV for NO and NO_2_, we can obtain the recovery time of the Zn_12_O_12_-based nanowire sensor at T = 300 K to be 1.5 μs and 16.7 μs, respectively, indicating the rapid response of the Zn_12_O_12_-based nanowire to NO and NO_2_ gases.

Experimentally, one of the most important parameters for sensing a gas is sensitivity (*S*), which is defined as follows:^74^

6 $$S=\exp (\frac{{E}_{g0}-{E}_{g}}{kT}),$$

where *k* is the Boltzmann’s constant and *T* is the temperature. *E*_g0_ is the band gap of the Zn_12_O_12_-based nanowire with gas adsorption, and *E*_g_ is the band gap of the pure Zn_12_O_12_-based nanowire. It is obvious that the sensitivity would grow exponentially with a decrease in *E*_g_. Yuan *et al*.^74^ have investigated the sensitivity of the ZnO surface using the above equation, which has been modified by taking account of environmental influence. The sensitivities of H_2_, NH_3_ and ethanol at 573 K they estimated are in agreement with experimental data. Although there are no experimental data that can used to compare with our calculated results, and the GGA-PBE calculations underestimate the band gap widths of semiconductors, we can estimate the trend of sensitivities of different molecules on the Zn_12_O_12_-based nanowire at room temperature. It can be concluded that the sensitivities of NO and NO_2_ gases are much larger than that of other gases because of the larger difference value of band gaps of the nanowire before and after adsorption of NO and NO_2_. This shows that the Zn_12_O_12_-based nanowire can be a highly sensitive gas sensor for NO and NO_2_ detection.

## Conclusions

In conclusion, using first-principles calculations based on density functional theory, firstly, we investigated the structural and electronic properties of cluster-assembled nanowires based on Zn_12_O_12_ cluster, then investigated the structures, energetics, charge transfer and electronic properties of the most stable Zn_12_O_12_-based nanowire adsorbed by environmental gases, including CO, NO, NO_2_, SO_2_, NH_3_, CH_4_, CO_2_, O_2_ and H_2_. Our results indicate that the ultrathin ZnO nanowires can be assembled by the coalescence of stable Zn_12_O_12_ cluster. The Zn_12_O_12_-based nanowires have semiconducting properties with direct energy gaps, and are particularly stable at room temperature. The CO, NO, NO_2_, SO_2_, and NH_3_ molecules are all chemisorbed on the Zn_12_O_12_-based nanowire with reasonable adsorption energies, but CH_4_, CO_2_, O_2_ and H_2_ molecules are only physically adsorbed on the nanowire with small *E*_ads_ and little charge transfer. The electronic properties of the Zn_12_O_12_-based nanowire present dramatic changes after the adsorption of the NO and NO_2_ molecules, especially their electric conductivity, however, the other molecules adsorption hardly change the electronic properties of the nanowire. Furthermore, The adsorption of NO (or NO_2_) molecule introduces spin polarization in the Zn_12_O_12_-based nanowire with a magnetic moment of approximately 1 *μ*_*B*_, indicating that magnetic properties of the Zn_12_O_12_-based nanowire is changed obviously due to the adsorption of NO (or NO_2_) molecule. Meanwhile, the strong adsorption of SO_2_ and NH_3_ makes their recovery times be much longer than 12 hours, which precludes its applications for SO_2_ and NH_3_ sensors, however, the recovery time of the nanowire sensor at T = 300 K is 1.5 μs and 16.7 μs for NO and NO_2_ molecules, respectively. Furthermore, the sensitivities of NO and NO_2_ are much larger than that of the other molecules. Therefore, synthetically considering the adsorption energies, charge transfer, the change of electric conductivity and magnetic properties, sensitivities and recovery time, we can conclude that the Zn_12_O_12_-based nanowire is a potential candidate for gas sensors with highly sensitivity for NO and NO_2_.

## Electronic supplementary material

Supporting information
