# Supplementary material for: The Zn12O12 cluster-assembled nanowires as a highly sensitive and selective gas sensor for NO and NO2
Source: Sci Rep. 2017 Dec 13;7:17505. doi: 10.1038/s41598-017-17673-8 (PMC5727522; doi:10.1038/s41598-017-17673-8)
Supplement: Supplementary file 1 — Supporting information [file 41598_2017_17673_MOESM1_ESM.doc]

**Supporting information**

**The** **Zn_12_O_12_ cluster-assembled nanowires as a highly sensitive and selective gas sensor for NO and NO_2_**

Yongliang Yong,[[1]](#footnote-2)*,1,2 Xiangying Su,^1,2^ Qingxiao Zhou,^1,2^ Yanmin Kuang,^2^ and Xiaohong Li^1,2^

^1^College of Physics and Engineering, Henan University of Science and Technology, Luoyang 471023, People’s Republic of China

^2^Henan Key Laboratory of Photoelectric Energy Storage Materials and Applications, Henan University of Science and Technology, Luoyang 471023, People’s Republic of China

^3^Institute of Photobiophysics, School of Physics and Electronics, Henan University, Kaifeng 475004, People’s Republic of China


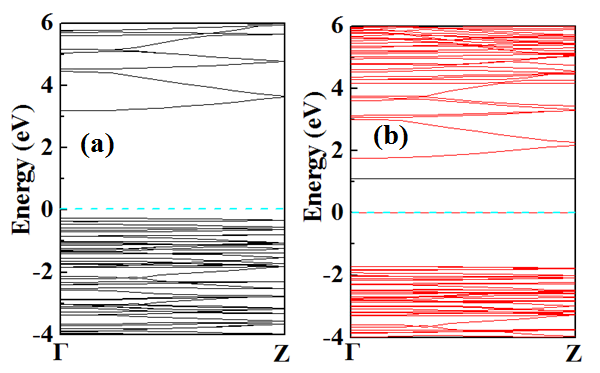


**Figure S1**: Calculated electronic band structures of (a) the Zn_12_O_12_-based nanowire without and (b) with NO adsorption using DFT-HSE06 method.

1. ^^Corresponding author.

   E-mail address: ylyong@haust.edu.cn [↑](#footnote-ref-2)
